# Study on Dynamic Mechanical Properties of Low-Alloy, High-Strength Steel Weld Metal at High Temperatures

**DOI:** 10.3390/ma18071488

**Published:** 2025-03-26

**Authors:** Liang Song, Yun Peng, Haiyan Zhao, Yang Cao, Lin Zhao

**Affiliations:** 1 School of Materials Science and Engineering, Tsinghua University, Beijing 100084, China; 2 Central Iron & Steel Research Institute, Beijing 100081, China

**Keywords:** low-alloy, high-strength steel, weld metal, high-temperature strength, dynamic mechanics, tensile strength, yield strength

## Abstract

To investigate the dynamic mechanical properties of low-alloy, high-strength steel weld metal at high temperatures, the temperature distribution equation and boundary conditions of weld metal during welding were determined. A steady-state temperature distribution model of weld metal heat loss was constructed by combining the heat loss equation and the heat source loss equation. Subsequently, a weld with Q960E high-strength steel extruded plate was used as the base material to investigate the dynamic mechanical properties of the weld metal at high temperatures. The experimental results show that the weld seam is the weakest region of the whole welded joint, and with the increase in temperature, the yield strength and tensile strength of the welded joints decrease. Heat treatment technology at high temperatures can significantly change the weld tensile strength of Q960E high-strength steel, and solid solution + aging treatment can optimize the mechanical properties of the heat-affected zone. We observed the short-term persistence of high-temperature metal at 600 °C/199 MPa and 650 °C/118 MPa; except for one 118 MPa short-term endurance test at high temperature, most samples qualified. With the increase in annealing temperature from 830 °C, the yield strength and tensile strength of the samples decreased significantly, and elongation after break increased dramatically.

## 1. Introduction

For manufacturers, to enhance production efficiency, φ5.0 mm heat-resistant steel welding rods are extensively used in the repair welding of low-alloy steel castings and product joint welding [1]. Regarding Q960E high-strength steel [2], the welding current of a φ5.0 mm electrode provided by welding material manufacturers is merely a reference value. Considering the lack of certification on the impact of high-current welding on joint performance, it is necessary to investigate the current limit of large-size welding rods. During the welding process, operations are carried out by using currents that exceed the conventional range. This unconventional current will significantly increase the heat input and impact the performance and material microstructure of the welded joint. However, large welding current inevitably results in significant heat input, and high temperature affects the performance of products after welding. However, there is limited research on the extent of damage caused by high temperatures to the mechanical properties of welded joints (particularly under high-temperature working conditions) and whether significant changes in the microstructure have occurred for such low-alloy weldments. Therefore, studying the change law of mechanical properties of welded joints by using this type of welding material at high temperature has significant practical implications, enabling the avoidance of adverse effects in actual production [3].

Gündoğdu et al. [4] conducted fiber laser cladding on 6061T6 aluminum alloy and performed mechanical and metallurgical analysis. By using a fiber optic laser, full penetration self-welding was achieved under −4 mm laser energy and −95 kW focusing conditions. Furthermore, the protective gas concentration varied for different stroke rates of molten pools. Macros and micrographs of materials at different rates were collected to evaluate the mechanical properties of materials (hardness, tensile, bending, etc.). The results indicated protective gas amounts and welding speeds significantly influenced weld quality. The mechanical properties diminished due to the coarsening of the precipitated phase. This method showed advantages of smaller molten pool width and heat-affected zone compared with conventional processes. Singh et al. [5] investigated the regulatory effect of strengthening methods on material microstructure by combining microstructural analyses. The size and distribution of strengthened particles in different parts of the FSW process were studied by using OM, SEM, and TEM. Currently, the processing zone (i.e., the melting zone) is a primary research concern, related to grain size and microstructure. Advanced research studies [6] focused on the spiral seat ring welding of Francis flow turbines. The seat ring (90 mm thickness) and spiral shell (36 mm) were welded. Actual construction may take months or years, and welding numerical simulation calculations may take months. Based on ANSYS software (2025 R1 v25.1) and APDL language, thermal stress coupling simulation analysis was performed for large-structure volute seat ring welding parts. Three simulation models were established, and the optimal scheme was selected based on blind hole method experimental data. Yan M. et al. carried out steady-state tensile tests on butt-welded specimens with artificial defects to investigate the effect of defects on the mechanical properties of butt welds at elevated temperatures. The effects of these defects on the stress and strain evolution patterns, fracture strength, and elongation capacity were discussed. The results showed that stress concentration due to defects in the weld zone reduces the yield strength of the weldment at elevated temperatures, and the stress concentration induces a low level of brittle fracture in the weld zone at elevated temperatures with different failure modes. The elongation capacity of weldments at different temperatures is strongly influenced by the degree of defects [7]. According to Qi K. et al., it is important to explore the effect of the heat treatment process on the properties of welded joints. Three different heat treatment processes were applied to welded joints with three different properties. Then, hardness tests, impact tests, tensile tests, and metallographic analysis were used to evaluate the performance of welded joints after heat treatment. The differences in mechanical properties for different combinations of welding processes and post-welding heat treatments were derived [8].

Although some progress has been made in the analysis of welding processes and material microstructures in existing research, there are still some shortcomings. At present, most research focuses on theoretical analysis and process design, lacking specific experimental data on the changes in mechanical properties of weld metal at different high temperatures, especially in quantitative research on the effects of heat treatment processes and annealing temperatures on weld metal properties. This study delves into the influence of these factors on the dynamic mechanical properties of weld metal, providing an important basis for optimizing material selection and welding processes.

## 2. Dynamic Mechanical Analysis of Weld Metal Based on Thermodynamic Equation

The establishment of thermodynamic equations can describe thermodynamic phenomena such as heat transfer and material phase transition in the welding process, thereby enabling more accurate prediction of the properties and behaviors of materials during welding. When investigating the dynamic mechanical properties of low-alloy, high-strength steel weld metal at high temperatures, it is essential to consider the influence of temperature gradient, thermal stress, phase transition, and other factors in the welding process on the weld. Establishing thermodynamic equations allows for the numerical simulation and prediction of these effects, providing a basis for subsequent experiments and analyses. The temperature distribution equation and boundary conditions of weld metal during welding are as follows:

(1) $$\frac{K}{c\rho }\frac{{\dot{p}}^{2}T}{\dot{p}e^{2}}+j^{2}\frac{\rho \prime }{c\rho }+v\frac{\dot{p}T}{\dot{p}e}=\frac{ℵ}{c\rho r}\left(T-T_{f}\right),$$

where c and ρ are the specific heat capacity and density of the weld metal, respectively; v and ℵ are the welding speed and the surface radiation coefficient, respectively; r and $\rho_{\mathrm{HRB}400}$ are the radius and the resistivity of the weld metal, respectively; K and j are the thermal conductivity and the current density, respectively [9]; T and $T_{f}$ are the welding temperature and the ambient temperature respectively; e is the welding energy; and $\dot{p}$ is the creep strain rate.

In Formula (1), $\frac{K}{c\rho }$ is heat conduction, $\frac{{\dot{p}}^{2}T}{\dot{p}e^{2}}$ is convection, $v\frac{\dot{p}T}{\dot{p}e}$ is internal heat source, $j^{2}\frac{\rho \prime }{c\rho }$ is the heat loss caused by air convection, and $\frac{ℵ}{c\rho r}\left(T-T_{f}\right)$ is the heat loss caused by heat radiation [10,11]. Accurately determining the loss of heat is helpful in understanding the direction of energy during the welding process and is crucial to analyzing changes in the welding temperature field. Heat loss and heat source loss are expressed by Formula (2):

(2) $$h\left(T-T_{f}\right)-\sigma ℵ\left(T^{4}-T_{f}^{4}\right)/r=ℵ\left(T-T_{f}\right)/r,$$

where B is the convection heat loss coefficient of weld metal and M is the heat dissipation coefficient of Stefan–Boltzmann law.

We calculate the critical value of heat flux distribution using internal heat sources. By determining this critical value, the critical boundary of heat transfer during the welding process can be clarified, and it can be known under what heat flux conditions the heat transfer characteristics of the welding process will undergo significant changes. Based on this, the critical value of heat flux distribution is calculated by using the internal heat source:

(3) $$K\frac{\partial T}{\sigma v\partial h}=\frac{\dot{p}T}{\dot{p}e},$$

We calculate the heat exchange between the steel plate boundary and the weld metal. Clarifying this heat exchange situation can provide a deeper understanding of the heat transfer law among different components during the welding process, which is of great significance for accurately simulating the distribution of the welding temperature field and analyzing the thermal influence relationship between the weld metal and the base metal. Combined with the thermal conductivity, the heat exchange between the steel plate boundary and the weld metal [12] is calculated as follows:

(4) $$K\frac{\partial C^{\prime }}{\partial h}=\alpha T^{\prime },$$

where α represents the external heat transfer coefficient of the weld zone of the steel plate and $T^{\prime }$ represents the temperature of the material around the weld zone.

We establish the relationship equation between strain and displacement in the weld zone and link the deformation caused by heat transfer with mechanical properties. According to the heat exchange model between the steel plate boundary and the weld metal, the relationship equation between strain and displacement in the weld zone is established as follows:

(5) $$\partial h\left\{d\varepsilon \right\}=\left[B\right]\left\{d\delta \right\}K\partial ℵ,$$

where $\left[B\right]$ represents the strain between material elements in the weld zone and the displacement vector matrix of the stress node and $\left\{d\varepsilon \right\}$ represents the connection between strain and displacement.

The primary cause of yield strength stress [13,14] and deformation in the welded area of a steel structure is excessive local heat input caused by uneven heating. Welded yield strength stresses mainly form due to thermal deformation and structural changes leading to phase transitions. Factors affecting the temperature field are the welding speed and the heat source. Consequently, in the process of calculating the temperature field of the welding area [15], it is necessary to fully consider the key factors affecting the calculation results of the temperature field. We set ℜ to represent the degree of heat source inside the thick steel plate, ∂ to represent the coefficient factor of the calculation of the temperature of the thick steel plate, and $\left(x,y,z\right)$ to represent the coordinates of the calculation point of the thick steel plate. We establish the relationship equation between strain and displacement in the weld zone to relate the deformation due to heat transfer to the mechanical properties, give a formula for the total control of heat transfer in the temperature field, calculate the temperature field in the weld zone more comprehensively and accurately, and ensure that the effects of various factors on the temperature distribution can be fully taken into account when simulating the welding process. According to the above analysis, the calculation formula for the total control of heat conduction in temperature field is as follows:

(6) $$c\rho \frac{\partial T}{\partial \tau }-ℜ-\frac{ℵ\partial }{\partial z}\left(K\frac{\partial T}{\partial z}\right)=\frac{\partial }{\partial y}\left(K\frac{\partial T}{\partial y}\right)+\frac{ℵ\partial }{\partial x}\left(K\frac{\partial T}{\partial x}\right),$$

where $\left(x,y,z\right)$ represents the coordinate point of the heat source center of the thick steel plate.

Based on this, the actual size of the weld pool [16,17] is obtained; then, the heat source model is corrected. Due to differences in steel plate implementation techniques and personnel capabilities, a dual ellipsoid heat source model was used to calculate the temperature field of the steel plates. By using a double-ellipsoid heat source model to calculate the temperature field of the steel plates, we clarify the relationship between heat flux and the energy density of thick steel plate heat sources, as well as the melt pool coefficient in the weld zone. The double-ellipsoid heat source model can better fit the distribution characteristics of heat sources in actual welding processes and more accurately simulate the energy transfer and distribution of welding heat sources in the steel plates. The calculation formula of the double-ellipsoid heat source model is as follows:

(7) $$q\left(x,y,z\right)=ℜ\prime \prime \cdot \exp\left(-\frac{y^{2}}{{a^{\prime \prime }}^{2}}\right)\cdot \exp\left(-\frac{x^{2}}{\rho^{2}}\right)\cdot \exp\left(-\frac{z^{2}}{c^{2}}\right),$$

where q represents the heat flux, ℜ″ represents the energy density of the heat source of the thick steel plate, and a″ represents the weld zone pool coefficient.

Given that the temperature field is formed by heat input to the weld zone, characterized by localized concentration, its distribution is highly inhomogeneous and typically unstable, leading to large deformations and stresses in the weld zone. To address heat input in various situations, two models—the double-ellipsoid heat source model and the Gaussian heat source model—are selected to calculate the temperature field of yield strength stress. In the actual calculation process, the appropriate model can be chosen to enhance the accuracy of the final numerical simulation. The adaptive range of the Gaussian heat source model is manual arc welding, tungsten–argon arc welding, and other welding methods [18,19]. It belongs to the normal distribution heat source model, affecting only the welding area. Consequently, only the working area will transfer heat energy to the steel plate, specifically the heating spots, and the Gaussian function is represented by the heat flux in the heating spots.

Based on different welding process parameters, we accurately calculate the heat flux in the Gaussian heat source model, so as to simulate the heat input and distribution during the welding process more accurately for specific welding methods. Therefore, the expression for setting the heat flux q is

(8) $$q\prime \prime =\frac{\eta UI}{q\left(x,y,z\right)Sv},$$

where η stands for the arc thermal efficiency of the thick steel plate, U stands for the voltage, I stands for the current, S stands for the cross-section area of the bridge weld zone, and v stands for the welding speed of the welding surface.

Clarifying the specific value of heat flux at the center of the heating spot during the welding process is of great significance for in-depth research on the thermal effects of welding heat sources on local areas, as well as analyzing the microstructure and mechanical property changes of welded joints at local locations. According to the above formula, the heat flux $q_{s}\left(r\right)$ between the heat source and the heating spot center of the thick steel plate during welding is calculated, and the formula is as follows:

(9) $$q_{s}\left(r\right)=\frac{q\prime \prime \Phi }{\pi r_{0}^{2}}e^{\frac{-r^{2}}{r_{0}^{2}}},$$

where r represents the heating spot center, Φ represents the power of the input heat source during the welding of the thick steel plate, and $r_{0}$ represents the actual radius of the input heat source.

It is assumed that the yield strength stress in the weld zone of the steel plate is along the long axis and uniformly distributed in the same thickness. Consequently, in the layer-by-layer milling process, when the thickness of the removed welding material is t, the actual thickness of the welding area of the thick steel plate is h; after stripping, the thickness of the remaining welding area is h−t, and the remaining welding part is deformed under the action of force and moment [20]. If the strain in the weld zone is ε, the calculation equation for the tensile strength of the weld metal is as follows:

(10) $$\phi \left(t\right)=q_{s}\left(r\right)\int \frac{\varepsilon \left(h-t\right)}{\left(h-z\right)}dz-E\frac{d\varepsilon }{dt},$$

where E represents the elastic modulus of the welding area.

In order to more accurately study the dynamic mechanical properties of low-alloy, high-strength steel weld metal at high temperatures, a finite element model is introduced for numerical simulation. The finite element model can effectively deal with complex geometries and boundary conditions and accurately analyze the temperature field and stress field in the welding process. The basic idea of the finite element model is to discretize the continuous solution domain into a combination of finite units, mechanically analyze each unit, and then combine the units to obtain the mechanical response of the whole structure. The finite element model adopts tetrahedral elements with a mesh density of 20 elements/mm^3^ to ensure the accurate simulation of complex geometric shapes in the welding area. The boundary conditions are set as follows: On the outer surface of the welding area, we define the thermal convection boundary conditions, with a convective heat transfer coefficient of 10 W/(m^2^·K) and an ambient temperature of 25 °C. In terms of mechanical boundary conditions, we fix the bottom of the welded specimen and simulate the constraint situation during actual welding. The material properties are set as follows: elastic modulus of 70 GPa, Poisson’s ratio of 0.33, and thermal conductivity of 200 W/(m·K). In order to verify the accuracy of the model, the results obtained from the simulation were compared with the experimental data, and the maximum error between the simulated temperature and the measured temperature was no more than 10 °C; the error between the simulated results and the experimentally measured stress value was within 5%, which proved that the model could accurately simulate the physical phenomena in the welding process.

In the welding process, for the temperature field analysis, the finite element method will be the discretized solution domain, and the temperature distribution within each unit can be obtained by the interpolation of the shape function. The relationship between the temperature T at any point within the unit and the node temperature $T_{i}$ is

(11) $$T=\sum_{i=1}^{n}N_{i}T_{i},$$

Among them, $N_{i}$ is a shape function, which is a coordinate function that depends on the shape of the elements and the distribution of nodes.

In the process of solving the temperature field, the above equation is substituted into the energy conservation equation and integrated and discretized on each unit. According to the Galerkin method, the finite element equation system can be obtained as

(12) $$\left[C\right]\left\{\dot{T}\right\}+\left[K\right]\left\{T\right\}=\left\{Q\right\},$$

Among them, $\left[C\right]$ is the heat capacity matrix; $\left[K\right]$ is the conduction matrix; $\left\{\dot{T}\right\}$ is the derivative vector of the node temperature with respect to time; $\left\{T\right\}$ is the node temperature vector; $\left\{Q\right\}$ is the heat flow of node heat.

For the stress field analysis, according to the mechanical equilibrium equation, under the assumption of small deformation, the stress–strain relationship in the finite element model can be expressed as

(13) $$\left\{\delta \right\}=\left[D\right]\left(\left\{\varepsilon \right\}-\left\{\varepsilon_{0}\right\}\right),$$

Among them, $\left\{\delta \right\}$ is the stress vector; $\left[D\right]$ is the elasticity matrix, which is related to the elastic modulus and Poisson’s ratio of the material; $\left\{\varepsilon \right\}$ is the total strain vector; $\left\{\varepsilon_{0}\right\}$ is the initial strain vector.

The relationship between the strain vector $\left\{\varepsilon \right\}$ and the displacement vector $\left\{u\right\}$ is determined through geometric equations:

(14) $$\left\{\varepsilon \right\}=\left[B\right]\left|\left\{u\right\}\right.,$$

Among them, $\left[B\right]$ is a geometric matrix composed of the derivatives of shape functions with respect to the coordinates.

By substituting the above equation into the mechanical equilibrium equation and discretizing it, the finite element equation system for solving the stress field can be obtained as

(15) $$\left[K_{s}\right]\left\{u\right\}=\left\{F\right\},$$

Among them, $\left[K_{s}\right]$ is the structural stiffness matrix, $\left\{u\right\}$ is the nodal displacement vector, and $\left\{F\right\}$ is the nodal force vector, including the external force and the equivalent nodal force due to thermal stresses caused by temperature changes. With the above finite element model, the dynamic mechanical properties of low-alloy, high-strength steel weld metal at high temperatures can be studied and analyzed more in depth during the welding process by combining the established thermodynamic equations and boundary conditions.

## 3. Experimental Design

### 3.1. Materials and Methods

#### 3.1.1. Experimental Materials

In the experimental procedure, a Q960E high-strength steel plate was selected as the base material (substrate), with dimensions of 400.0 mm × 200.0 mm × 15.0 mm, and the filling material was MG70-G gas-shielded welding wire with a diameter of 1.2 mm. This selection was based on the high strength of Q960E high-strength steel. Its yield strength can reach 960 MPa, which is suitable for high-load and high-stress working environments. It also has excellent mechanical properties, such as high tensile strength and good toughness. Additionally, it shows certain thermal stability in normal working temperature ranges. MG70-G gas-shielded welding wire exhibits good compatibility with Q960E high-strength steel. It can facilitate the formation of high-quality and uniform welds. This welding wire possesses high strength and toughness, which can ensure that the welded joint has good mechanical properties. Furthermore, during the welding process, MG70-G gas-shielded welding wire has good fluidity and wettability, which allow it to effectively fill the weld seam and form a uniform welding structure. The combination of these materials satisfies the research requirements.

**Figure 1:**
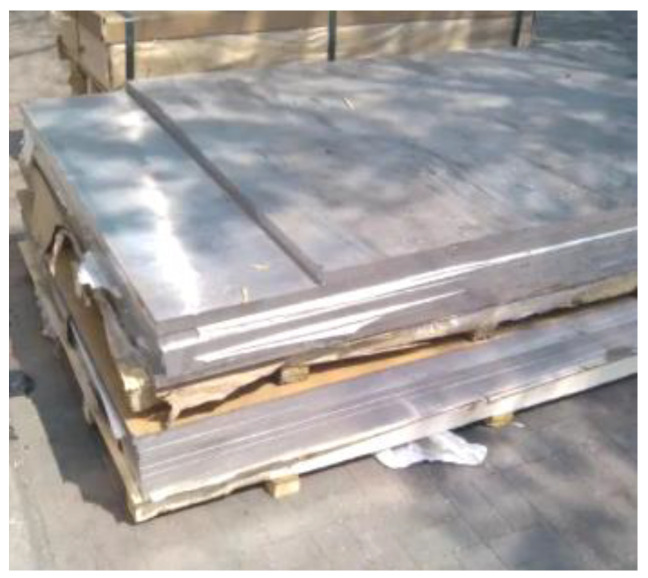
Q960E high-strength steel plate.

**Figure 2:**
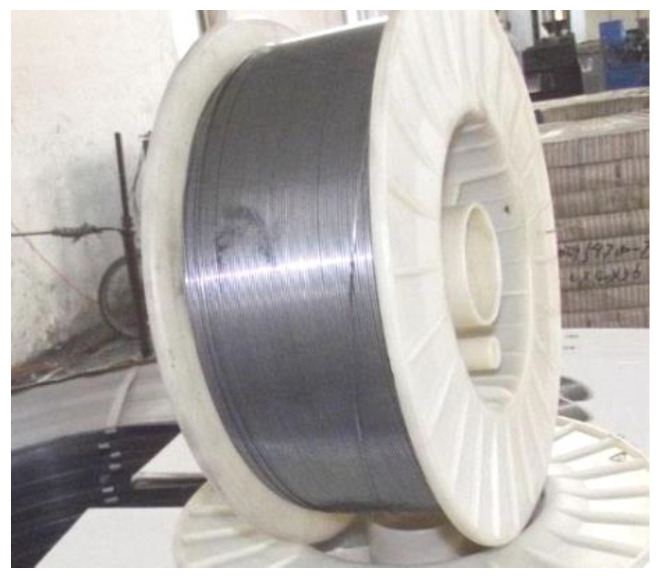
MG70-G gas-shielded wire.

Given the welding type for the melting electrode of gas-shielded welding (MIG) and the welding equipment selection of a full digital control pulse MIG welding machine, the equipment has a stable current output and good welding performance. The shielding gas is a mixture of 98% argon (Ar) and 2% carbon dioxide (CO_2_), which can effectively protect the welding area, reduce oxidation, and help stabilize the arc. The welded joints are designed as butt joints, with a single V-type bevel, a bevel angle of 60°, a passive edge thickness of 2 mm, and a root gap of 3 mm, to ensure the good fusion and molding of the weld during the welding process. By using a Q960E high-strength steel plate as the base material and MG70-G gas-shielded welding wire for welding, the aim is to ensure high quality and efficiency of the welding process, thereby meeting the stringent requirements for the study of dynamic mechanical properties of low-alloy, high-strength steel weld metal at high temperatures. The test specimens of the Q960E high-strength steel plate and MG70-G gas-shielded wire are shown in Figure 1 and Figure 2.

The chemical composition and mechanical properties of the substrate and welding wire are shown in Table 1 and Table 2.

#### 3.1.2. Experimental Methods

Two heat treatment techniques, solution + aging and aging treatments, were selected for the experiment. The process parameters for solution + aging were as follows: the sample underwent a solid solution process at 550 °C for 3 h, followed by rapid water quenching and subsequent transfer to a heat treatment furnace at 180 °C for 8 h. For the aging treatment, the sample was maintained at 180 °C for 8 h.

With optical microscopy, we examined the metallographic structure of the welded joint at various positions, and an electronic universal experimental machine with a loading rate of 12 mm/min conducted the tensile test. A Vickers hardness tester was used to test the hardness of the welded joint. The test positions ranged from the center of the weld to the side of the base material, with a 3 mm interval between adjacent measurement points. In order to investigate the microstructural characteristics of the weld metal of low-alloy, high-strength steel at high temperatures in depth, SEM technology was used to carry out detailed microstructural analysis (Fremont, CA, USA). By using SEM, the surface microstructure of the weld metal was observed at high resolution, with SEM clearly showing grain morphology, size distribution, and the microcharacteristics of defects such as porosity and cracks in the weld area and providing an intuitive basis for the understanding of the microstructure of weld metal at a larger scale. For scanning electron microscopy analysis, we used a JSM-7800F scanning electron microscope (Tokyo, Japan) with a magnification set to 500–5000 times, adjusted according to observation needs. The sample was etched by using etchant for 30 s to clearly display the microstructure. Image analysis was performed by using ImageJ software (1.54k), which manually outlined grain boundaries to measure grain size and calculate the average grain area and grain size distribution. For optical microscope analysis, we used a SAGA microscope with a magnification of 100–400 times (Shenying Optics Co., Ltd., Suzhou, China). The etching agent was the same as for the SEM analysis, and the etching time was 20 s. We observed grain morphology and distribution and the presence of defects in optical microscope images.

FLUKE 572-2 was employed to measure the welding metal and the surrounding temperature of steel (Fluke Testing Instruments (Shanghai) Co., Ltd., Shanghai, China). It is a handheld high-temperature infrared thermometer for applications including steel welding at 1000 °C, capable of measuring temperatures from −30 °C to 900 °C with high accuracy. When using an infrared thermometer for high-temperature steel welding, one needs to align it with the weld area and press the measurement button. During measurement, the distance and angle between the thermometer and the measurement target must be kept consistent to ensure that no objects obstruct the thermometer’s field of view to ensure the accuracy and reliability of the measurement results. When measuring, one needs to align the measuring end of the thermometer vertically with the weld metal surface or heat treatment area and keep the measuring distance of 10 cm and the measuring angle of 90°. Temperature data are to be recorded every 30 s to ensure continuity and accuracy. Potential sources of error include ambient light interference, oxidized layers on the measurement surface, and roughness effects. In order to minimize the interference of ambient light, a light shield was set up around the measurement area; for the oxidized layer on the measurement surface, sandpaper was used to gently sand down the oxidized layer to remove it before measurement; for the effect of roughness on the surface, an area with a relatively flat surface was selected for measurement.

We chose a lower load of 150 gf to avoid excessive indentation affecting test accuracy or damaging the material surface. The indentation time was set to 20 s. If the time is too short, the indentation may not be fully formed, and the hardness measurement value may be higher; if the time is too long, the material may experience creep and other phenomena, resulting in low hardness measurement values.

The dimensions of the tensile specimen were a length of 50 mm, a length of 60 mm, a width of 12.5 mm, a thickness of 3 mm, and a strain rate of 0.001 s^−1^; the strain was measured by an electronic extensometer, and the accuracy of the extensometer was ±0.001 mm. The data of the load and displacement were collected at a frequency of 10 times per second to ensure the completeness and accuracy of the test data.

### 3.2. Experimental Feasibility Verification

**Figure 3:**
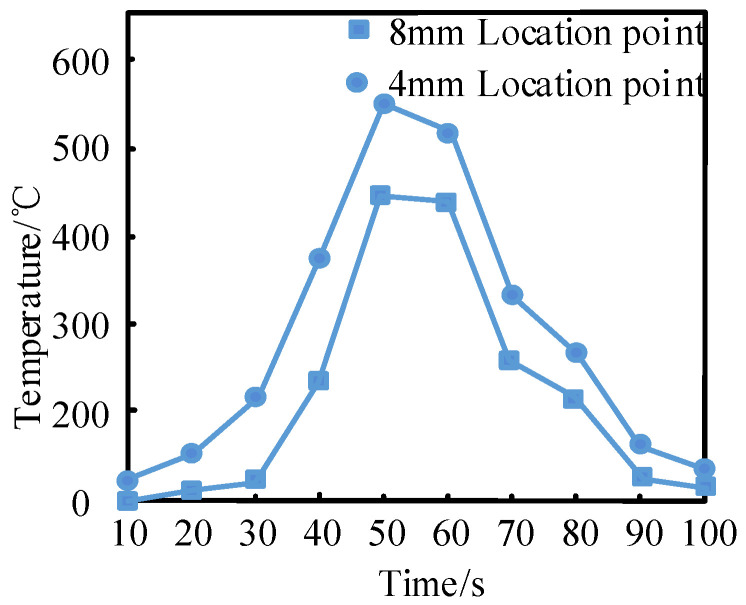
Temperature field simulation of welding process.

The temperature field changes in the welding process were simulated at the distances of 4 mm and 8 mm on both sides of the weld center, and the results are shown in Figure 3.

As observed in Figure 3, the temperature at 4 mm and 8 mm on both sides of the weld increased as welding progressed. At the conclusion of welding at 50 s, the temperature at 4 mm and 8 mm reached the maximum values of 550 °C and 430 °C, respectively, before decreasing. Notably, the temperature at 4 mm from the weld was always higher than that at 8 mm. The simulation demonstrates that temperature increases in proximity to the weld center and decreases with the increase in distance. After 50 s, the temperature at both 4 mm and 8 mm from the weld center declined gradually, with the temperature at 4 mm decreasing more rapidly than at 8 mm. The weld cooled to approximately ambient temperature after 100 s. The simulation results align with empirical observations, indicating that the experimental design is both feasible and reliable.

## 4. Results and Discussion

### 4.1. Test of Fracture Position of Tensile Specimens at High Temperatures 

In the investigation of dynamic mechanical properties of high-strength steel weld in high-temperature environments, examining the fracture position of tensile specimens under high-temperature conditions allows for the evaluation of material toughness and brittleness. This assessment predicts the material’s service life in practical engineering applications. Table 3 presents the normal temperature mechanical properties of joints at different high temperatures, with all tensile specimen fractures occurring in the weld.

Table 3 demonstrates that the weld represents the weakest region of the entire welded joint. As the temperature increases, the yield strength and tensile strength of the joint exhibit a decreasing trend. Based on the experimental results, the fracture characteristics and mechanism of the material can be preliminarily analyzed and the failure causes and patterns investigated, potentially providing insights for material design and engineering applications. Furthermore, by examining the fracture location under different conditions, the high-temperature mechanical properties of different materials or treatment methods can be compared, offering data support for material selection and engineering.

### 4.2. Tensile Properties

When investigating the dynamic mechanical properties of high-strength steel welds in a high-temperature environment, analyzing the tensile properties of welded joints enables the evaluation of their strength and stability. The tensile properties of welded joints are related to their deformation and failure characteristics. Analyzing these characteristics in the tensile process provides deep insights into the mechanical properties and failure mechanism of welded joints.

#### 4.2.1. Tensile Properties of Welded Joints Under Different Heat Treatments 

**Figure 4:**
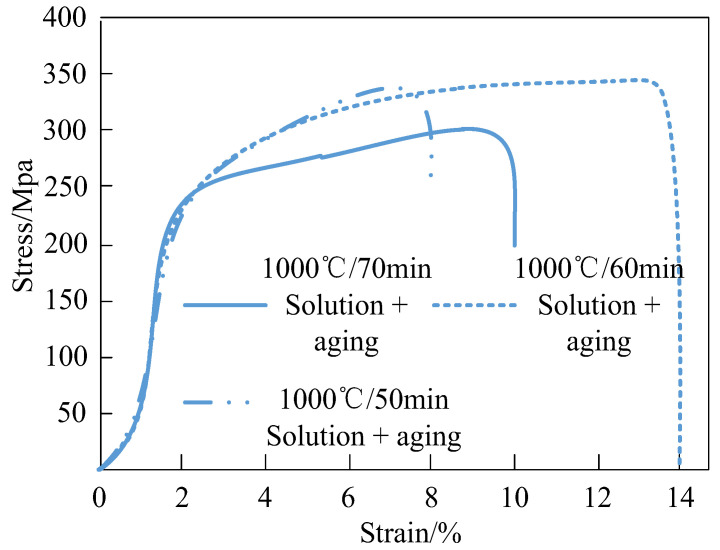
Stress changes in the sample under different process parameters.

Heat treatment can modify the microstructure of Q960E high-strength steel, altering its tensile strength through effects on the weld (Baoshan Iron and Steel Group Co., Ltd., Shiyan, China). Changes in the microhardness of the welded joint following heat treatment directly influence its tensile strength. Figure 3 illustrates the stress changes in the sample after tensile treatment using heat treatment technology under the process parameters of 1000 °C/50 min, 1000 °C/60 min, and 1000 °C/70 min. The results are shown in Figure 4.

As can be seen from Figure 4, heat treatment technology can significantly change the welding tensile strength of Q960E high-strength steel. But the temperature field simulation has certain limitations. The model assumes that the heat transfer in the welding process is ideal, ignoring factors such as uneven heat radiation and the nonlinearity of the material thermophysical properties with temperature change that may exist in actual welding.

#### 4.2.2. Analysis of Tensile Fracture of Welded Joint

The welded joint of Q960E high-strength steel was used as the experimental base material, with six parts selected as samples. Specimens from a to c are specimens in the welding state, and those from d to f are specimens after solution + aging treatment. The mechanical experiment was conducted according to GB-2651 [21]. It specifies the preparation of test specimens and testing procedures for the transverse tensile testing of welded butt joints, with the aim of determining their tensile strength and fracture location, and is applicable to the transverse tensile testing of all welded butt joints. The results are shown in Table 4.

By analyzing Table 4, it can be seen that the yield strength of the original base material is 3150 MPa, the tensile strength is 280 MPa, and the elongation is 13%. In the welded state, for the three samples a, b, and c, the yield strength is between 1960 and 2000 MPa, the tensile strength is between 122 and 127 MPa, the elongation is between 16 and 17%, and the fracture location is in the heat-affected zone, which is due to the softening of the heat-affected zone by the welding thermal cycle. After solid solution + aging treatment, the yield strength of the three samples d, e, and f is between 2800 and 3100 MPa, the tensile strength is between 133 and 135 MPa, the elongation is between 11 and 12%, and the fracture location is in the weld zone, which indicates that the solid solution + aging treatment optimizes the mechanical properties of the heat-affected zone so that the yield strength is close to that of the original substrate and improves the mechanical properties of the welded joints.

### 4.3. Influence of High-Temperature Strength on Short-Time Durability of Welded Joints at High Temperatures

When studying the dynamic mechanical properties of high-strength steel welds in a high-temperature environment, the short-term durability of welded joints is crucial to evaluating their mechanical properties. In high-temperature environments, welding joints are susceptible to softening and failure, which affect their short-term durability. Appropriate annealing treatment can enhance the grain boundary and intracrystalline structure of welded joints, improving their high-temperature stability and short-term durability. The service life and performance of welded joints in high-temperature environments can be determined by analyzing the change law of the short-time lasting performance of welded joints under different conditions.

Although the high-temperature strength of the welded test plates under several high-temperature conditions meets the minimum requirements of the base metal, a high-temperature short-term durability test was conducted on the above welded joints to more accurately evaluate their performance under actual working conditions. The test duration was 2 h to simulate the short-term high-temperature exposure under actual working conditions. The criteria for determining qualification were as follows: Under the conditions of 600 °C/199 MPa and 650 °C/118 MPa, if there was no obvious deformation, crack propagation, or fracture of the welded joint within 2 h, it was judged as qualified; if any of the above failure phenomena occurred, it was judged as unqualified. The specific definition of the failure criterion is as follows: when visible cracks appear on the surface of the welded joint and the crack length exceeds 2 mm, if the deformation of the joint exceeds 5% of the original size, it is considered that the welded joint has failed. The experimental results are shown in Table 5.

High-temperature short-term endurance tests at 600 °C/199 MPa and 650 °C/118 MPa were performed on the welded joints. All tests were successful except for the lower half of the 6 # test plate, which failed under 650 °C/118 MPa conditions (double tests failed). In the high-temperature short-term durability test, only one of the ten samples failed at 650 °C/118 MPa. From the perspective of the material itself, the sample may present, at the microscopic level, impurities or grain growth abnormalities, where impurities will become stress concentration points, under the action of high temperature and stress, and the main cause of crack expansion and grain growth abnormalities make the distribution of grain boundaries not uniform and reduce the strength of the grain boundaries, weakening the overall load-bearing capacity of the material. This failure sample provides important clues for exploring the weakness of the material, and through its in-depth analysis, we can pinpoint the potential problems in the microstructure, welding process, and heat treatment of the material, so as to improve the material formulation, optimize the welding process, perfect the heat treatment process, and improve the comprehensive performance and reliability of the material in high-temperature environments.

### 4.4. The Effect of the Annealing Temperature on the Structure of the Heat-Affected Zone

Temperature is a critical factor influencing the microstructure and properties of high-strength steel weld metal. The heat-affected zone (HAZ) of high-strength steel welds is the area impacted by heat during welding, affecting welding quality and service life. The optimal annealing temperature can be determined by analyzing the microstructure variation in the HAZ at different annealing temperatures. The faster the welding speed, the smaller the area of the heat-affected zone (HAZ) as the material is exposed to the heat source for a shorter period of time, which minimizes unfavorable microstructural changes and preserves the mechanical properties of the base material to a greater extent. Conversely, slower welding speeds result in larger heat-affected zones and more pronounced microstructural changes, which may lead to the softening of the heat-affected zone and the degradation of the mechanical properties. Annealing temperature refers to the temperature in the heat treatment process of metal materials, where the material is heated, maintained, and then slowly cooled to alleviate internal stress and enhance structure and performance. At the annealing temperature, the grain boundary and the intracrystalline structure of the metal material will change, affecting the mechanical properties, electrical properties, and other physical properties of the material. Different metal materials and application requirements necessitate varying annealing temperatures and durations to attain optimal performance. Generally, the annealing temperature correlates with the melting point and grain size of the metal; materials with higher melting points and larger grain sizes require higher annealing temperatures.

**Figure 5:**
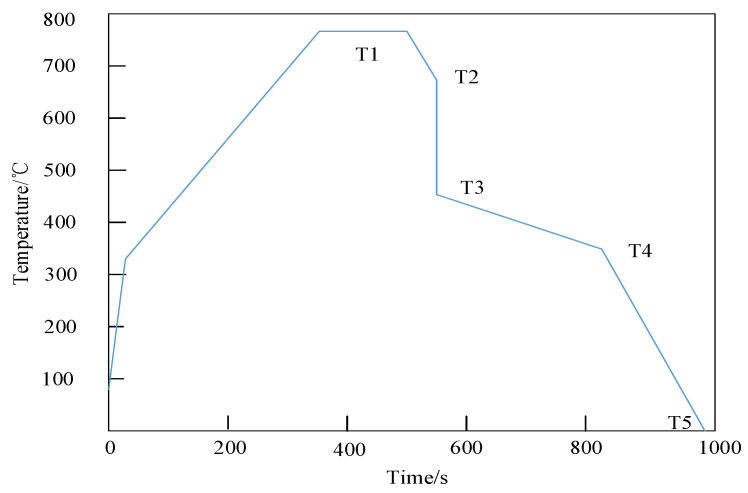
Control curve of continuous annealing process.

Figure 5 shows the process control curve simulating the continuous annealing process.

Figure 5 shows that during the continuous annealing process, T1 is the isothermal annealing temperature, at which the internal atoms of the material obtain sufficient energy to diffuse, eliminate some internal stresses, and homogenize the structure. T2 is the slow cooling temperature, and controlling the cooling rate can affect grain growth and microstructure transformation; a slower cooling rate is beneficial to forming coarse and uniform grain structures. T3 is the rapid cooling temperature, which can suppress the precipitation of certain unfavorable phases and obtain a specific microstructure. T4 is the over-aging temperature, which can further grow and aggregate the precipitated phase and adjust the mechanical properties of the material. T5 is the final cooling temperature, which cools the material to room temperature to complete the entire annealing process. The time of each stage is precisely controlled based on the material properties and experimental objectives to achieve the optimization of the microstructure and properties of the material. During rapid cooling, the undercooling of the weld metal is high, the nucleation rate is high, but the growth rate is limited; a fine-grain structure can easily form, which increases the hardness and strength of the weld but reduces its toughness. When slowly cooled, atoms have enough time to diffuse, which is conducive to the formation of a coarse-grain structure. At this time, the strength and hardness of the weld are lower, but the plasticity and toughness are better.

The soaking annealing temperatures in this experiment were set to 830, 850, 860, 870, 880, 890, 900, 910, and 930 °C. This is because, in the Q960E high-strength steel plate, in this temperature interval, recrystallization, grain growth, and other important structural changes occur. When the annealing temperature is lower than 830 °C, the recrystallization process is incomplete and will retain part of the work-hardening organization; when the temperature is higher than 930 °C, the grain will be overgrown, resulting in a decline in mechanical properties. The annealing holding time was set to 2 h, which gives the atoms enough time for diffusion and recrystallization to optimize the mechanical properties of the weld metal. The tensile test machine was used to test the mechanical properties of the samples annealed at different temperatures, and the microstructure was observed by an optical microscope.

**Figure 6:**
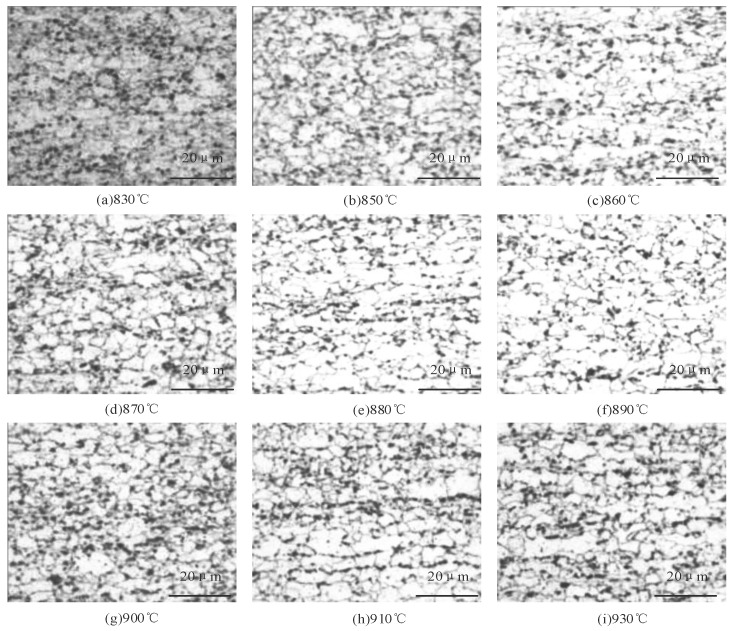
Metallographic structure at different annealing temperatures.

Figure 6 shows the metallographic structures of the samples, all of which are ferrite and pearlite structures, at different annealing temperatures.

Figure 6a demonstrates that at 830 °C, some banded and incompletely recrystallized microstructures remain. At 850 °C, the partially recrystallized strip structure largely disappears, but grain size is non-uniform with some smaller grains present (Figure 6b). As the annealing temperature continues to increase, the number of the smaller grains in the microstructure gradually decreases. At 890 °C, smaller grains are essentially eliminated, and grain polygonization is substantially complete, with a grain size of about 6 μM. From Figure 6f–i, no significant change in grain size is observed as the annealing temperature further increases, due to the pinning effect of Nb precipitates hindering grain growth.

### 4.5. Effect of Annealing Temperature on Mechanical Properties

**Figure 7:**
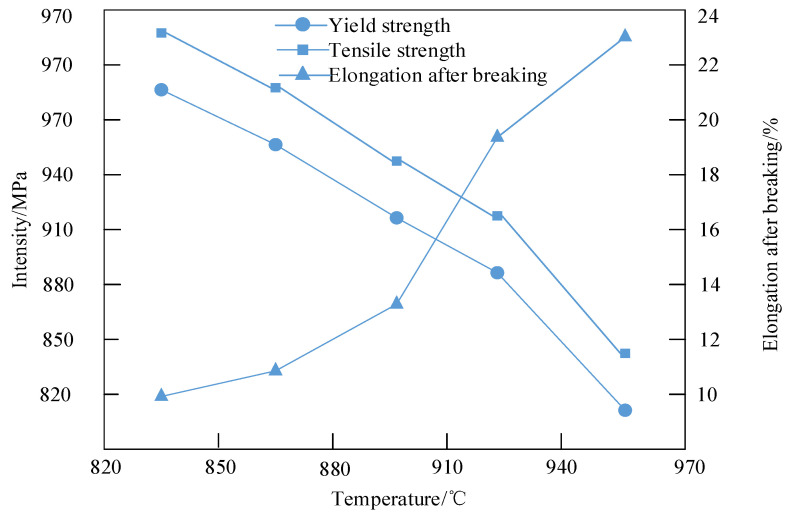
Relationship between annealing temperature and mechanical properties of annealed samples.

By analyzing the changes in mechanical properties of high-strength steel weld metal at different annealing temperatures, the optimal annealing temperature for the best mechanical performance can be determined. Furthermore, in high-temperature environments, high-strength steel weld metal is susceptible to softening and failure, which adversely affects its mechanical properties. Appropriate annealing treatment enhances the grain boundaries and intragranular structure, improving mechanical properties at high temperatures. Figure 7 illustrates the relationship between annealing temperature and the mechanical properties of the annealed samples.

Figure 7 shows that below 890 °C, an increase in annealing temperature corresponds to a significant decrease in yield strength and tensile strength, with a substantial increase in elongation after fracture. This is due to the considerable microstructural changes, including recrystallization and grain polygonization. The reduction in work hardening from cold rolling decreases yield strength and tensile strength while increasing elongation after fracture. Above 890 °C, the yield strength and tensile strength of the sample continue to exhibit a decreasing trend with the increase in annealing temperature, but more gently and stably. Figure 6f reveals that at 890 °C, smaller grains have largely disappeared, indicating completed recrystallization and polygonization. At higher temperatures, the grain size remains stable, but Nb precipitates coarsen or decompose, reducing precipitation strengthening and gradually decreasing yield strength and tensile strength. Thus, the decrease in yield strength and tensile strength above 890 °C is attributed to weakened precipitation strengthening rather than grain growth.

Different welding techniques have a significant impact on research results. Laser welding with high energy density and small heat input can make the weld heat-affected zone narrow and reduce the influence on the properties of the base material; the weld organization is finer and more uniform, which can improve the strength and toughness of the joints, but it may produce cracks due to the excessive cooling speed. Stir friction welding, a solid-phase welding method, to avoid some of the defects of melting welding, can refine the grain, improve the microstructure of the joint, and improve the mechanical properties and its forging organization to make the joint strong, and plasticity and fatigue performance is good. The cooling speed has a key influence on the microstructure of the weld zone. With rapid cooling, a large degree of subcooling, a high nucleation rate, but a limited growth rate, fine-grain structures easily form, such as martensite or bainite, with higher hardness and strength but lower toughness; slow cooling is conducive to the diffusion of atoms and the formation of coarse-grain structures (such as pearlitic or ferrite-pearlitic structures), and while strength and hardness are lower, plasticity and toughness are better. The geometry of the joint affects research results. In terms of joint design, butt joints have uniform stress distribution, which is favorable to withstanding tensile forces, but require high assembly accuracy; lap joints are easy to assemble, but the stress concentration is obvious, which reduces the joint bearing capacity. Constraints change the stress–strain state during the welding process, and increased constraints increase the welding residual stress, which may lead to cracks and affect the performance and life of the joint. Prolonged exposure to high temperatures changes the properties of weld metal. Atomic diffusion exacerbates the growth or coarsening of the precipitated phase, weakening the effect of precipitation strengthening and reducing strength and hardness; high temperature may also trigger elemental diffusion and recrystallization, changing the microstructure, reducing the fatigue properties and creep properties of materials, and affecting the long-term reliability of the welded structure in high-temperature environments, so the study of the impact of long-term high-temperature exposure on material behavior in practical application is of great significance.

We validated the results of the analysis of how welding speed affects the width of the heat-affected zone and the mechanical properties of the weld for a given material. We analyzed the optimum welding speeds to minimize the adverse effects of welding on the surrounding material while maximizing the mechanical strength and ductility of the weld. The results are shown in Table 6.

Analysis of Table 6: As the welding speed increases from 10 mm/s to 30 mm/s, the width of the HAZ decreases dramatically, from 6.5 mm to 2.5 mm. The decrease in the width of the HAZ indicates that the faster the welding speed, the lower the heat input and the stronger the local thermal effect, which minimizes the alteration in the microstructure and the potential weakening of the material in the HAZ. At the same time, the mechanical properties of the welds improve with the increase in welding speed. The yield strength increases from 850 MPa at 10 mm/s to 920 MPa at 30 mm/s, and the tensile strength increases from 900 MPa to 960 MPa. Similarly, elongation at break, a measure of ductility, increases from 12 percent at 10 mm/s to 15 percent at 30 mm/s. This shows that faster welding speeds not only minimize adverse thermal effects on the surrounding material but also increase the mechanical properties of the weld, thus improving overall weld quality and performance.

## 5. Conclusions

Low-alloy, high-strength steel is widely used in aviation, aerospace, automotive and mechanical sectors, and other fields, with many applications requiring materials to maintain strength and toughness in high-temperature environments. Consequently, investigating the dynamic mechanical properties in high-temperature environments can provide valuable guidance for optimizing material selection, improving welding processes, and enhancing high-temperature strength. The experiment obtained the following results:

(1) The weld seam is the weakest area of the welded joint. As temperature increases, the yield strength and tensile strength of the welded joint decrease.
(2) The heat treatment technology at high temperatures can significantly alter the welding tensile strength of high-strength steel. Solid solution + aging treatment can significantly optimize the mechanical properties of the heat-affected zone of welded joints.
(3) In the high-temperature short-term endurance tests at 600 °C/199 MPa and 650 °C/118 MPa, the high-temperature short-term endurance of the specimens was assessed. It can be assumed that within a certain range of swing width, the slower the welding speed in the forward direction, the longer the high-temperature residence time at a specific point (such as 800–1200 °C), resulting in greater relative high-temperature intensity. When swing width is excessive, the welding rod point heat source takes longer to return to a point after high temperature, and the temperature may cool below 800 °C. Although the welding speed is slower in the forward direction, the total high-temperature residence time does not increase linearly with the increase in swing width (in extreme cases, it can be considered transverse welding).
(4) According to the micrograph of the metallographic structure of the welded joint when the annealing temperature is 830 °C, with the gradual increase in the annealing temperature, the yield strength and tensile strength of the sample decrease significantly, while the elongation after fracture increases substantially.

In the welding structure of the engine compartment, due to the high temperature generated during engine operation, traditional welding processes may lead to a decrease in the strength of the welded joints at high temperatures, affecting the reliability of the structure. Based on the findings of this study, it was found that the weld seam is the weakest area of the welded joint, and the yield and tensile strength of the joint decrease as the high-temperature strength increases. When designing the welding process, engineers can refer to research to adopt appropriate solid solution + aging treatment for welded components, optimize the mechanical properties of the heat-affected zone of the welded joint, improve the strength and stability of the welded joint at high temperatures, and ensure the safety of the engine compartment structure in high-temperature working environments. Long-term exposure to high temperatures can cause significant changes in the mechanical properties and microstructure of welded joints. Atomic diffusion intensifies, and the precipitation phase in the weld grows and coarsens, resulting in the weakening of the effect of precipitation strengthening, welded joints, strength, and hardness; at the same time, elemental diffusion and recrystallization changes in the microstructure occur, so that the grain grows and grain boundaries are weakened, reducing the fatigue properties of the material and creep performance.

In terms of research limitations, although this article strictly controls the experimental conditions, the complex working conditions in actual production may lead to deviations between experimental results and practical applications. And this research study mainly focuses on short-term high-temperature performance, with insufficient research on the performance evolution and failure mechanism of welded joints under long-term high-temperature service conditions. In future research directions, it is recommended to conduct more direct experimental studies on steel to obtain more accurate performance data on steel welds at high temperatures, combine advanced microscopic characterization techniques, conduct in-depth research on the detailed mechanism of microstructure evolution of steel at high temperatures, consider the coupling effects of multiple factors under actual working conditions, and establish a more realistic experimental model to provide a more reliable theoretical basis for the engineering application of low-alloy, high-strength steel in high-temperature environments.

## Figures and Tables

**Table 1:** Chemical composition of substrate and welding wire (%).

Mass Fraction	Materials	
	6082	ER5087
Si	0.10	0.05
Fe	0.18	0.13
Cu	0.03	<0.02
Mn	0.61	1.06
Mg	0.96	4.73
Cr	0.05	0.11
Zn	0.02	<0.02
Ti	0.03	0.09
Al	Margin	Margin

**Table 2:** Mechanical properties of substrate and welding wire.

Index	Materials	
	6082	ER5087
Yield strength, MPa	301	159
Tensile strength, MPa	341	288
Elongation at break, %	14.8	17.7

**Table 3:** Effects of different high temperatures on mechanical properties of joints at normal temperature.

Plate Number	Maximum Heat Input, (kJ/cm)	Tensile Strength, MPa	Yield Strength, MPa	Maximum Impact Value of Weld, J		
				Heat-Affected Zone	Welded Joint	Matrix Region
1#	16.25	694	581	157	102	208
2#	20.55	617	571	144	124	214
3#	35.97	603	560	152	116	226
4#	41.78	594	552	159	108	259
5#	56.88	580	557	120	114	237
6#	66.79	573	542	135	159	272
7#	72.11	569	531	137	147	254
8#	86.30	567	527.8	146	126	256
9#	96.71	557	436.9	158	120	248
10#	100.23	552	459.4	159	117	249

**Table 4:** Mechanical properties of joints after heat treatment.

Experimental Material		Yield Strength, MPa	Tensile Strength, MPa	Elongation, %	Fracture Location
Raw substrate		3150	280	13	-
Normal welded condition	a	2000	127	17	Heat-affected zone
	b	1960	123	16	Heat-affected zone
	c	1980	122	16	Heat-affected zone
State under solution + aging heat treatment	d	2800	133	12	Weld zone
	e	2950	135	11	Weld zone
	f	3100	135	11	Weld zone

**Table 5:** Experimental results of short-time durability and high-temperature stability under different heat inputs.

Argument	1#	2#	3#	4#	5#	6#	7#	8#	9#	10#
600 °C/199 MPa	Qualified	Qualified	Qualified	Qualified	Qualified	Qualified	Qualified	Qualified	Qualified	Qualified
650 °C/118 MPa	Qualified	Qualified	Qualified	Qualified	Qualified	Unqualified	Qualified	Qualified	Qualified	Qualified

**Table 6:** Statistical analysis experiments.

Welding Speed, (mm/s)	HAZ Width, mm	Yield Strength, MPa	Tensile Strength, MPa	Elongation at Break, %
10 (slow)	6.5	850	900	12
20 (moderate)	4.0	900	950	14
30 (fast)	2.5	920	960	15

## Data Availability

The data presented in this study are available on request from the corresponding author due to privacy or ethical restrictions.
